# Continuous Activation of the CD122/STAT-5 Signaling Pathway during Selection of Antigen-Specific Regulatory T Cells in the Murine Thymus

**DOI:** 10.1371/journal.pone.0019038

**Published:** 2011-04-26

**Authors:** Jérémie D. Goldstein, Robert S. Balderas, Gilles Marodon

**Affiliations:** 1 UPMC Université Paris 06, UMR 7211, Paris, France; 2 CNRS, UMR 7211, Paris, France; 3 INSERM, U959, Paris, France; 4 Becton Dickinson, San Diego, California, United States of America; University of Nebraska Medical Center, United States of America

## Abstract

Signaling events affecting thymic selection of un-manipulated polyclonal natural CD25^+^foxp3^+^ regulatory T cells (nTreg) have not been established *ex vivo*. Here, we report a higher frequency of phosphorylated STAT-5 (pSTAT-5) in nTreg cells in the adult murine thymus and to a lesser extent in the periphery, compared to other CD4^+^CD8^−^ subsets. In the neonatal thymus, the numbers of pSTAT-5^+^ cells in CD25^+^foxp3^−^ and nTreg cells increased in parallel, suggesting that pSTAT-5^+^CD25^+^foxp3^−^ cells might represent the precursors of foxp3^+^ regulatory T cells. This “specific” pSTAT-5 expression detected in nTreg cells *ex vivo* was likely due to a very recent signal given by IL-2/IL-15 cytokines *in vivo* since (i) it disappeared rapidly if cells were left unstimulated *in vitro* and (ii) was also observed if total thymocytes were stimulated *in vitro* with saturating amounts of IL-2 and/or IL-15 but not IL-7. Interestingly, STAT-5 activation upon IL-2 stimulation correlated better with foxp3 and CD122 than with CD25 expression. Finally, we show that expression of an endogenous superantigen strongly affected the early Treg cell repertoire but not the proportion of pSTAT-5^+^ cells within this repertoire. Our results reveal that continuous activation of the CD122/STAT-5 signaling pathway characterize regulatory lineage differentiation in the murine thymus.

## Introduction

Natural regulatory CD4^+^CD25^+^foxp3^+^ T cells (nTreg) are generated in the thymus of mice and humans early in their ontogeny [1], [2], [3]. These nTreg cells are in charge of controlling peripheral T cells that have escaped negative selection in the thymus and that could pose a threat to the integrity of healthy tissues. The importance of the transcription factor Foxp3 for controlling auto-immunity in humans is best exemplified in patients suffering from the IPEX (Immunodysregulation, Polyendocrinopathy, and Enteropathy, X-linked) syndrome, who develop a potentially lethal autoimmune syndrome and that carry mutations in the Foxp3 gene [4]. Furthermore, ectopic expression of foxp3 confers suppressive function to murine naïve T cells [5] whereas ablation of foxp3 in nTreg cells leads to severe autoimmunity [6], [7]. Similar cases of autoimmunity have been found in nTreg cells that have spontaneously lost foxp3 expression [8]. Thus, a better understanding of foxp3 regulation is needed for translating the huge therapeutic potential of nTreg cells to the clinic.

Signaling pathways that lead immature thymic progenitors to differentiate into foxp3-expressing cells began to be elucidated (recently reviewed in [9]). Strong experimental evidence point to a crucial role of the TCR signal in nTreg cell “commitment” in the thymus and notably of CARMA-1 [10], [11] and LAT [12] molecules. From TCR transgenic studies, it also appears that a higher affinity (and thus, presumably a higher signal) perceived by an immature T cell ultimately impacts on regulatory commitment, at least in terms of frequencies of CD4^+^CD25^+^ cells [13]. This enrichment in nTreg cell differentiation of antigen-specific cells upon encounter with the cognate antigen in the thymus was also observed for some [14] but not all [15], [16] specificities. The reason for the discrepancy in the results might be due to the existence of a “niche” for nTreg cell differentiation in the thymus. It was indeed elegantly shown that the size of the available nTreg “niche” is as important as the affinity of the TCR itself for efficient nTreg cell maturation [17], [18].

Besides TCR signals, cytokines have also been proposed to play an important role in nTreg cell “commitment” in the thymus. Before the identification of foxp3 as the most reliable marker for regulatory T cells, it was already noticed that CD4^+^CD25^+^ T cells presented a peculiar response to IL-2 signaling [19]. Studies using genetically deficient mice have expanded our knowledge on the role of IL-2 signaling in foxp3 expression but several caveats remain. Deletion of the ß chain of the interleukin-2 receptor (CD122) (which comprises two additional units CD25 and CD132 (IL-2R-gc)) resulted in a complete absence of foxp3^+^ cells in the thymus in some [20], [21], but not all studies [22]. In contrast to mice simply deficient for IL-2 or CD25 [22], [23], CD132-KO mice have a dramatic decrease in total number of T cells and a quasi-absence of foxp3^+^ cells [22]. Interestingly, a lack of foxp3^+^ cells was also observed in mice doubly-deficient for IL-2 and IL-15 (a cytokine that share CD122 and CD132 with IL-2) [20], suggesting that foxp3 expression in the thymus of IL-2-KO animals observed in earlier studies [22], [23] could be due to a compensatory action of IL-15. In that respect, a role for IL-15 in directing foxp3 expression in human cells has recently been proposed [24]. A further support for a role of IL-2 in driving foxp3 expression came from studies showing that enforced expression of STAT-5, a transcription factor downstream of CD122 and known to drive foxp3 expression [20], [25], leads to enhanced nTreg cell generation in the thymus [26], [27]. Based on these data and others [28], the current model of nTreg differentiation in the thymus postulate a two-step pathway in which TCR and co-stimulatory signals generate CD25^+^foxp3^−^ precursor cells, followed by a gc-mediated cytokine signal allowing full expression of foxp3 and generation of mature CD25^+^foxp3^+^ *bona fide* nTreg cells.

Here, we characterize the expression of pSTAT-5 in CD25^+^foxp3^+^ cells, either in a static view in adult mice, or in a more dynamic perspective during nTreg generation in the neonates or during negative selection induced by an endogenous Sag. Our results suggest that activation of the STAT-5 signaling pathway may play a more complex role during nTreg differentiation than the mere induction of foxp3 expression.

## Materials and Methods

### Mice

C57BL/6J Ly5.2 female mice and DBA/2 male mice were purchased from Janvier laboratories (Le Genest Saint Isle, France). C57BL/6×DBA/2 F1 or C57BL/6 neonates were generated in our animal facility. All animals were kept under specific pathogen-free conditions and manipulated according to European council directive 86/609/EEC. The study was approved by the Regional Ethical Comittee 3 of Ile-de-France (ref p3/2008/039).

### Flow cytometry analysis

C57BL/6×DBA/2 F1 or C57BL/6 neonates were sacrificed on ice and their thymus was removed. Adult mice were sacrificed by cervical dislocation. After mechanical dissociation and trypan blue exclusion counts, 10^6^ cells were labeled with 100 µl of a mix of mAbs diluted in PBS containing 3% FCS for 30 minutes at 6–8°C in the dark. When needed, biotin-labeled mAb against TCR were used alone, followed by a mix containing the FITC-labeled streptavidine and the other mAbs. Intracellular staining using foxp3 mAb was performed according to the kit manufacturer's instructions (eBioscience). For pSTAT-5 detection, cell suspensions were rapidly fixed after sacrifice or after in vitro culture in 10 volumes of a solution of PBS 1.5% formaldehyde for 10 minutes at room temperature. Cells were then washed in a solution of PBS containing 0.2% of BSA, and permeabilized with 100% methanol for 10 minutes on ice. After extensive washing with PBS 0.2% BSA, cells were incubated with the phospho-specific mAb in combination with the mAbs of interest for 30 minutes in the dark at room temperature. In some experiments, a pSTAT-5 (Y694) blocking peptide (AKAVDGpYVKPQIKQV) was used as a negative control (fig. S1). Otherwise, the pSTAT5 negative threshold was defined on unstimulated cells or on cells stained with all flurorescent antibodies minus pSTAT5. At least 100,000 events were collected on a FACS LSR II (Becton Dickinson, San Jose, CA). Data were analyzed using FlowJo software (TreeStar, Ashland, OR).

### *In vitro* cytokine stimulation

Cells from the thymus or from the spleen of C57BL/6 adult mice were stimulated in RPMI 1640 (InVitrogen, Cergy-Pontoise, France) supplemented with 10% FCS, 2 mM glutamine, 100 U/ml penicillin, 100 µg/ml streptomycin, 50 µM β-Mercaptoethanol and 10 mM HEPES with cytokines at 50 ng/ml of murine recombinant IL-15 (Peprotech, Paris, France) or 50 ng/ml human recombinant IL-7 (Miltenyi Biotec, Paris, France), or 10 to 50 ng/ml of murine recombinant IL-2 (Peprotech) for 30 to 60 minutes in a 37°C, 5% CO_2_ incubator. At the end of incubation, cell suspensions were fixed in 10 volumes of PBS 1.5% formaldehyde for flow cytometry analysis.

### Antibodies

The following mAbs and reagents were used for phenotypic and repertoire analysis: peridinin-chlorophyll-protein (PercP)-, Alexa fluor 700- or Pacific blue-labeled anti-CD4 (RM4-5, Becton Dickinson (BD), San Diego, CA), PercP-labeled anti CD8 (53-6.7, BD), Alexa fluor 700-labeled anti-CD8 (53-6.7, eBioscience, San Diego, CA, USA), PE-Alexa Fluor 610-conjugated anti-CD8 (53-6.7, Caltag Laboratories, Burlingame, CA, USA), PE-Cyanin 7-conjugated anti-CD25 (PC61, eBioscience), biotinylated and PE-labeled anti-CD122 (TMß1, BD), Pacific Blue-, eFluor 450- or Alexa fluor 700-labeled anti-foxp3 (FJK16s, eBioscience), PE- and biotin-labeled anti-BV6 (RR4-7, BD), PE- and biotin-labeled anti-BV11 (RR3-15, BD), PE- and biotin-labeled anti BV8.1/8.2 (MR5-2, BD), FITC- and PE-labeled streptavidine (BD). Alexa fluor 488- and Alexa fluor 647-labeled anti-pSTAT-5 (Y694) were from BD.

### Statistical analyses

Two-tailed unpaired *t* test with 95% confidence intervals were performed using GraphPad Prism version 5.0 for PC (GraphPad Software, San Diego, CA). Mean values were considered statistically different if the *p* value was below 0.05.

## Results

### Preferential STAT-5 phosphorylation in foxp3^+^ cells in adult mice

**Figure 1:**
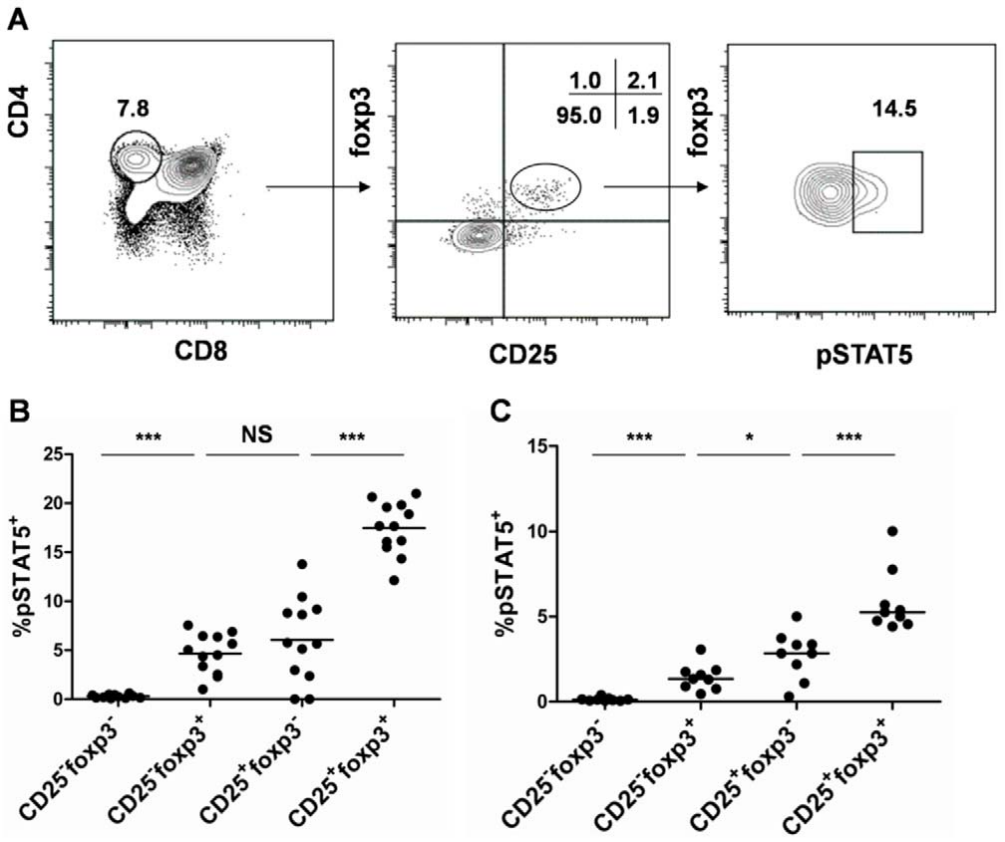
Preferential phosphorylation of STAT-5 in foxp3^+^ cells in adult C57BL/6 mice. (**A**) The gating strategy is indicated by the arrows. *Left panel*: total thymocytes were stained with CD4 and CD8 antibodies. The indicated gate defines CD4^+^CD8^−^ cells. *Middle panel*: CD25 and foxp3 expression in CD4^+^CD8^−^ cells. *Right panel*: pSTAT-5 expression in CD25^+^foxp3^+^CD4^+^CD8^−^ cells. (**B–C**) Frequencies of pSTAT-5^+^ cells within thymic CD4^+^CD8^−^ cells of the indicated CD25/foxp3 phenotype in the thymus (**B**) and in the mesenteric lymph nodes (**C**) in 6- to 13- week-old mice. Each dot represents a single mouse. Results are compiled from 4 to 11 independent experiments. The p values are indicated in each panel (***p<0.001; *p<0.05; NS not significant).

To investigate the association between pSTAT-5 and foxp3 expression in adult mice, we quantified the frequencies of pSTAT-5^+^ cells in various CD4^+^ subsets defined by CD25 and foxp3 expression in the thymus and in the periphery of adult C57BL/6 mice (fig. 1). In an attempt to preserve fleeting phosphorylation events occurring *in vivo*, we fixed freshly isolated thymocytes with formaldehyde (FA) few minutes after sacrifice. Cell suspensions were then stained with the antibodies detecting the CD4, CD8, CD25, foxp3 molecules and the phosphorylated form of STAT-5 at a Y in position 694. The specificity of the staining was validated using a pSTAT-5 blocking peptide, either on freshly isolated cells (fig S1A) or in vitro on IL-2-stimulated cells (fig S1B). A faint staining was detected for pSTAT-5 on freshly isolated FA-treated CD4^+^CD8^−^ thymocytes, mainly in foxp3^+^ cells (fig. 1A). We observed that the frequencies of pSTAT-5^+^ cells were the highest in CD25^+^foxp3^+^ cells, where up to 20% of the cells expressed pSTAT-5 (fig. 1B). The other subsets in which pSTAT-5 could be detected were CD25^−^foxp3^+^ and CD25^+^foxp3^−^ cells which bared a similar proportion of pSTAT-5+ cells.Of note, the median fluorescence intensities of pSTAT5 did not differ among the various subsets analyzed (fig S1C). Very low frequencies (<0.1%) of pSTAT-5^+^ cells were observed in CD25^−^foxp3^−^ cells, which comprise the majority of CD4^+^CD8^−^ cells of the thymus. Likewise, pSTAT-5^+^ cells were present in greater frequencies in CD25^+^foxp3^+^ cells in the peripheral lymph nodes albeit at much lower frequencies than in the thymus (fig. 1C). One possibility to explain these results might be that the total amount of STAT5 molecules differs in the various CD4 subsets defined by CD25 and foxp3 expression. To rule out this possibility, we measured the expression of STAT5 by flow cytometry in the various CD4 subsets in the thymus. Our results clearly show that STAT5 was homogeneously expressed and at similar levels in all the CD4+ subsets analyzed (fig S1), directly showing that the increased proportion of pSTAT5+ in CD4+CD25+foxp3+ cells was not due to a higher proportion of STAT5+ cells in this subset. Altogether, these results demonstrate directly for the first time that STAT-5 is preferentially phosphorylated in vivo within CD4^+^CD8^−^CD25^+^foxp3^+^ cells of the murine thymus.

### Kinetics of pSTAT-5 and foxp3 expression in the neonatal thymus

**Figure 2:**
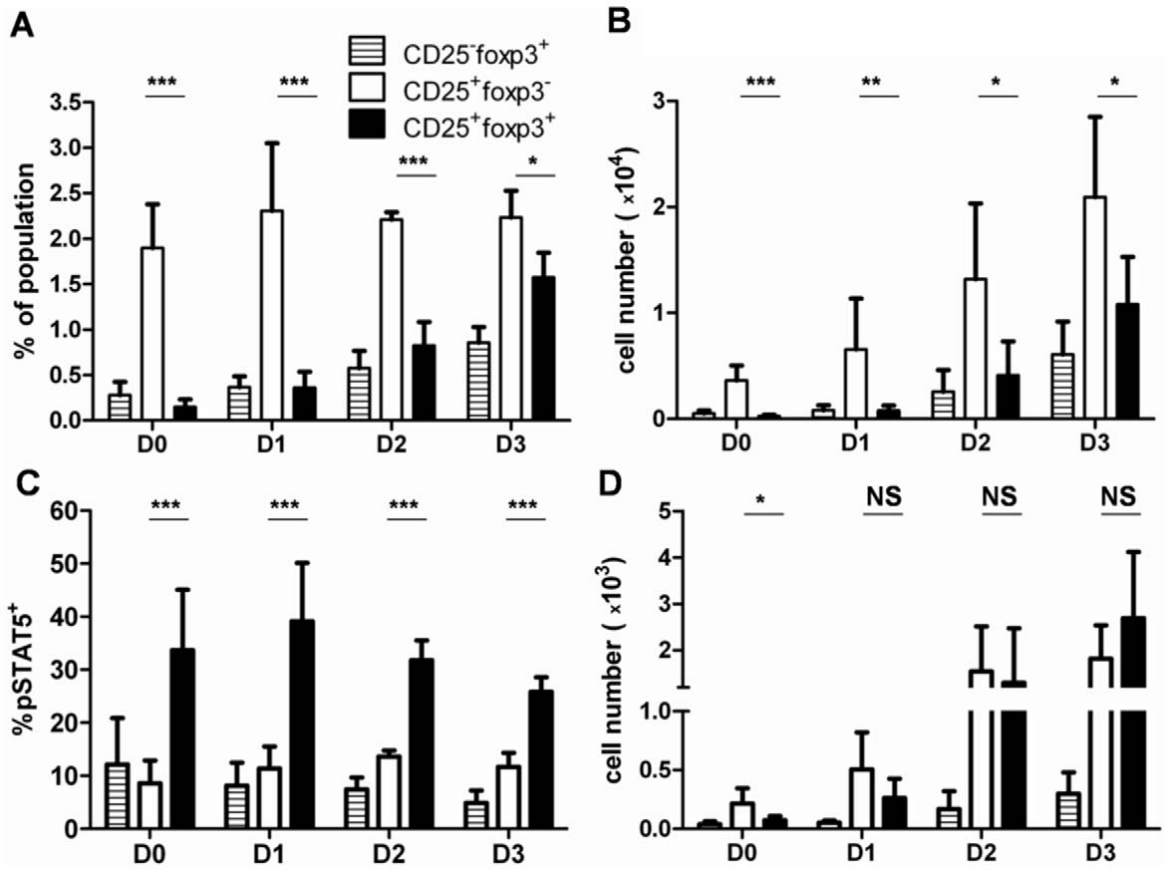
Kinetics of pSTAT-5 expression during Treg cell differentiation in the neonatal thymus of C57BL/6 mice. (**A–B**) Frequencies (**A**) and absolute numbers (**B**) of CD4^+^CD8^−^ cells of the indicated phenotype defined by CD25 and foxp3 expression in the thymus of C57BL/6 neonates at the indicated days after birth. For sake of clarity, analysis of CD25^−^foxp3^−^ cells which encompass >90% of all CD4^+^ T cells, is not included in the figure (**C–D**) Frequencies (**C**) and absolute numbers (**D**) of pSTAT-5^+^ cells within CD4^+^CD8^−^ thymic subsets defined by CD25 and foxp3 expression in C57BL/6 neonates at the indicated days after birth. The p values are indicated in each panel (***p<0.001; **p<0.01; *p<0.05; NS not significant). Results shown are the compilation of 2 independent experiments with 6 to 8 mice analyzed per day. For sake of clarity, only the p values comparing CD25^+^foxp3^−^ and CD25^+^foxp3^+^ cells are shown.

We then wanted to know whether pSTAT-5 and foxp3 expression would also be associated during early nTreg thymic differentiation. We first assessed the appearance of CD25^+^foxp3^−^, CD25^+^foxp3^+^ and CD25^−^foxp3^+^ subsets in the first 3 days of life in normal C57BL/6 mice (B6) (fig. 2A–B). At all time points analyzed, the CD25^+^foxp3^−^ cells represented the majority of the analyzed subsets, in frequencies and in numbers (fig. 2A–B, white bars). The numbers of CD25^+^foxp3^−^ cells were always higher than the numbers of the CD25^+^foxp3^+^ cells (fig. 2B), suggesting that only a fraction of CD25^+^foxp3^−^ cells may convert into CD25^+^foxp3^+^ cells. We then analyzed pSTAT-5 expression in these subsets (fig. 2C–D). We found a remarkably stable profile of pSTAT-5 expression in terms of frequencies in the analyzed subsets. More than 30% of CD25^+^foxp3^+^ cells expressed pSTAT-5 from birth, a slightly higher value than the one observed in adult mice. This frequency tended to decrease after D1 of life (fig. 2C, black bars), to reach similar values than the one observed in adult mice (fig. 1B). Expression of pSTAT-5 in other subsets was less abundant since it concerned less than 10% of the cells and no significant changes occurred during the period analyzed (fig. 2C). When numbers of pSTAT-5^+^ cells were considered, we observed a simultaneous increase in CD25^+^foxp3^−^ and in CD25^+^foxp3^+^ cells in the first 3 days of life (fig. 2D), consistent with the hypothesis that these two subsets are inter-related. Thus, our results show that pSTAT-5 is closely associated with foxp3 acquisition in the murine thymus early during ontogeny.

### Preferential STAT-5 phosphorylation in foxp3^+^ thymocytes *in vitro* correlates with CD122 but not CD25 expression

**Figure 3:**
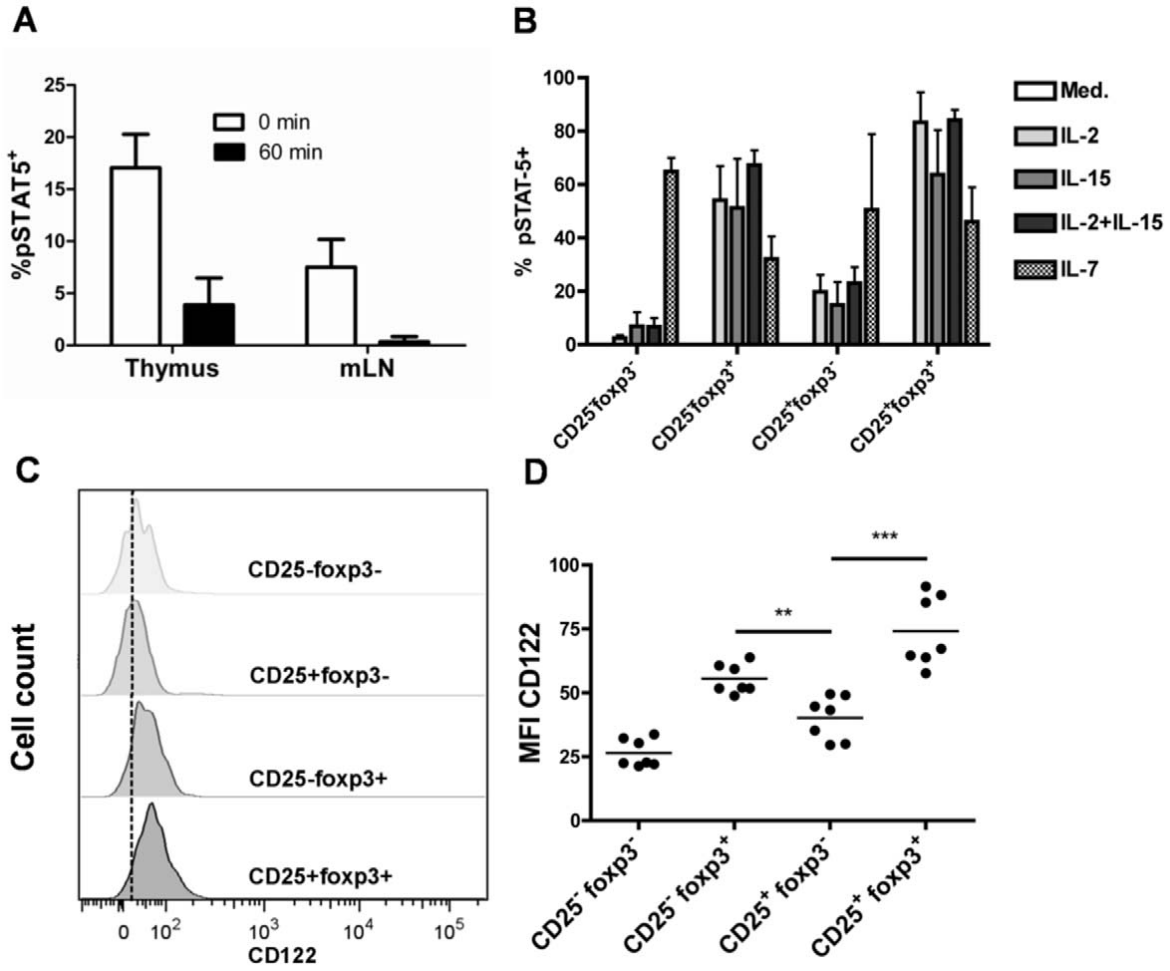
Preferential STAT-5 phosphorylation in foxp3^**+**^ thymocytes *in vitro* correlates with CD122 expression. (**A**) Frequency of pSTAT-5^+^ cells in CD25^+^foxp3^+^CD4^+^CD8^−^ cells of the thymus and of the mesenteric lymph node (mLN) fixed with formaldehyde immediately after sacrifice (0 min.) or after 60 minute incubation on ice (60 min.). (**B**) Frequency of pSTAT-5^+^ cells in thymocytes of the indicated CD25/foxp3 phenotype after *in vitro* incubation with the indicated cytokines for 60 min. (Med; medium only). Results shown are compiled from 2 to 8 independent experiments. (**C**) Representative profile of CD122 expression in the indicated thymic subsets defined by CD25 and foxp3 expression. Note the higher expression of CD122 in foxp3^+^ cells (**D**) Median fluorescence intensity of CD122 expression in thymic subsets defined by CD25 and foxp3 expression. Each dot represents a single mouse. Results shown are compiled from 2 independent experiments. The p values are indicated in the figure (***p<0.001; **p<0.01).

Interestingly, pSTAT-5 expression was detected at much lower frequencies if cells from the thymus or from the periphery were allowed to sit on ice for an hour before fixation and staining (fig. 3A), showing that the results gathered on freshly isolated cells likely reflected a very recent signal received *in vivo*. In an attempt to identify a molecule responsible for such a signal, we cultured freshly isolated thymocytes with molecules known to activate STAT-5 through CD132. Remarkably, IL-2 or IL-15 (or a combination of the two) preferentially activated STAT-5 in foxp3^+^ cells, whether the cells expressed CD25 or not (fig. 3B). Interestingly, CD25^−^foxp3^+^ but not CD25^+^foxp3^−^ cells were highly susceptible to IL-2/IL-15 stimulation *in vitro*. Only 15–20% of CD25^+^foxp3^−^ cells activated STAT-5 upon IL-2/IL-15 stimulation whereas 50–70% of CD25^−^foxp3^+^ cells were able to activate STAT-5 in response to these cytokines. In contrast to IL-2 or IL-15, IL-7 induced a high proportion of pSTAT-5^+^ cells in conventional CD25^−^foxp3^−^ cells whereas this cytokine induced pSTAT-5 only in a limited numbers of nTreg compared with Il-2 or Il-15 (fig. 3B). IL-4 and IL-21 did not induce STAT-5 phosphorylation on total thymocytes in our hands (data not shown).

The higher proportion of pSTAT-5^+^ cells in foxp3^+^ cells upon IL-2/IL-15 stimulation was most likely due to the higher levels of CD122 expression on the surface of foxp3^+^ cells compared to foxp3^−^ thymocytes (fig. 3C). Overall, CD25^+^foxp3^+^ cells had almost two-fold more CD122 molecules expressed at the cell surface than CD25^+^foxp3^−^ cells (fig. 3D). A similar pattern of expression was observed for CD132, with the highest expression found in foxp3^+^ cells (data not shown). Thus, the results suggest that IL-2 and/or IL-15 but not IL-7 are likely candidates for the enriched pSTAT-5 expression in CD25^+^foxp3^+^ cells that we detected *ex vivo*. They also show that foxp3 and CD122 but not CD25 expression correlated with greater susceptibility to IL-2 signaling *in vitro*.

### Enhanced selection of Mls-1^a^–specific T cells into the regulatory lineage in the thymus is not associated with increased proportions of pSTAT-5^+^ cells

**Figure 4:**
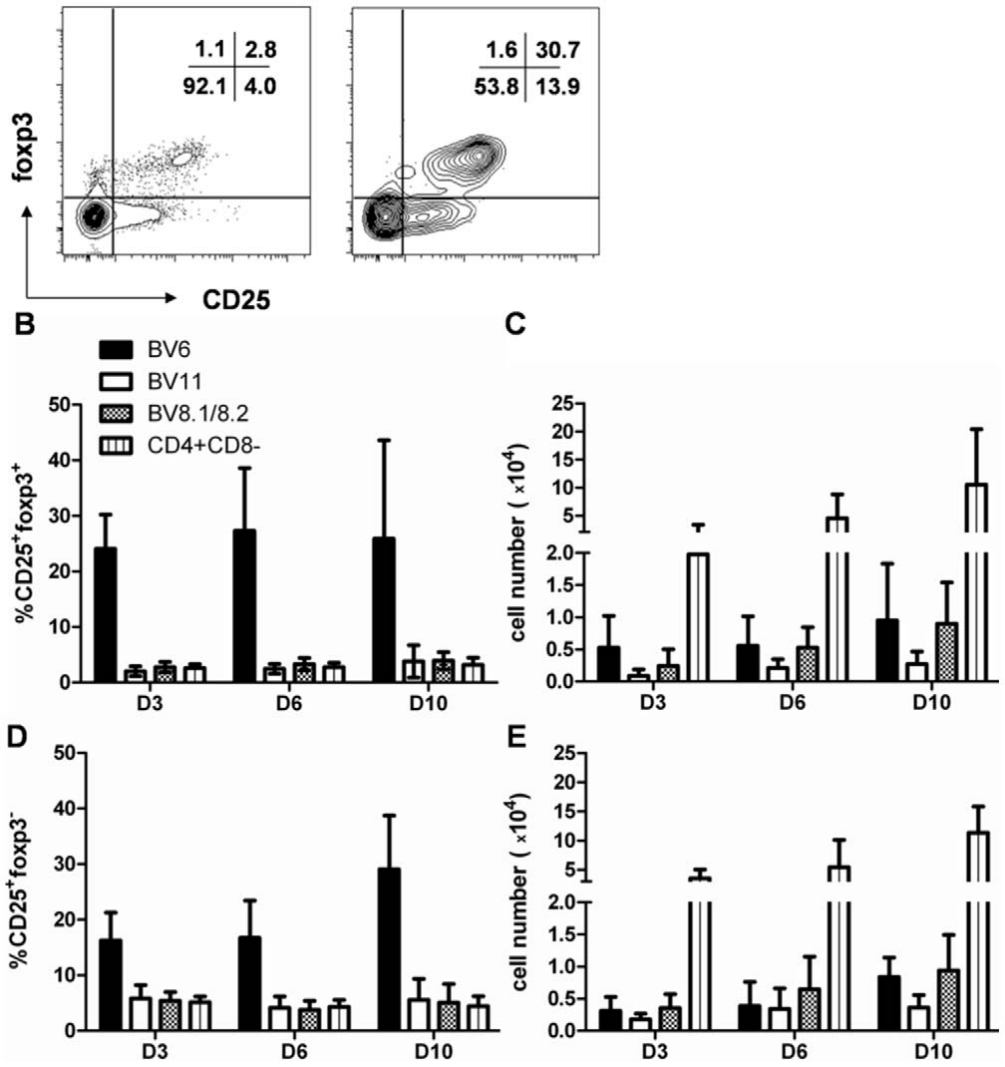
Enhanced selection of Mls-1^a^–specific T cells into the regulatory lineage in the thymus of C57BL/6**×**DBA/2 F1 neonates. (**A**) Representative flow cytometry analysis of CD25 and foxp3 expression in BV6^+^ or BV6^−^ cells among CD4^+^CD8^−^ thymocytes of a F1 mouse at day 3 after birth. (**B–E**) Frequencies and absolute numbers of CD25^+^foxp3^+^ (**B–C**) or CD25^+^foxp3^−^ cells (**D–E**) were determined in CD4^+^CD8^−^ cells within the indicated TCR-expressing cells (BV6, BV11, BV8.1/8.2) from 3 to 21 mice at the indicated age after birth in B6D2 mice. Results shown are compiled from 17 independent experiments.

Numerous studies in TCR transgenic mice have shown that nTreg selection is increased in the setting where forced expression of the cognate antigen is directed in the thymus. We thus wanted to know whether negative selection of self-specificities within a polyclonal repertoire would also be characterized by an increase in selection of nTreg expressing the same specificity. For this, we studied TCR-induced negative selection of BV6^+^ T cells in the neonatal thymus of C57BL/6×DBA/2 F1 mice (B6D2) due to recognition of the endogenous superantigen Mls-1^a^ [29]. As a control, we quantified the frequencies of BV6^+^ cells in the thymus of neonatal B6 mice. As expected, the frequencies of BV6^+^ T cells determined in CD4^+^CD8^−^ cells of the thymus decreased 2.5-fold during the first 10 days of life of B6D2 mice while the distribution of other specificities within CD4^+^CD8^−^ cells remained stable (fig. S2). We observed a marked increase in the frequencies of CD25^+^foxp3^+^ cells within Sag-reactive cells compared to the rest of the BV6^−^CD4^+^CD8^−^ thymocytes at day 3 after birth (fig. 4A). Overall, frequencies of “mature” CD25^+^foxp3^+^ T cells in BV6^+^ T cells were increased at every time point compared to BV11^+^CD4^+^, BV8.1/8.2^+^CD4^+^ or total CD4^+^CD8^−^ cells (fig. 4B). This increase was due to the expression of the Mls-1^a^ Sag since it was not observed in aged-matched B6 mice (fig S3A–B)). These enhanced frequencies translated in numbers of BV6^+^ of the CD25^+^foxp3^+^ phenotype that were comparable to other specificities unaffected by the Sag, such as BV8+ cells (fig. 4B), showing that negative selection (as defined by physical elimination) of Mls-1^a^-specific Treg cells was not apparent. A similar observation was made for nTreg “precursors” i.e CD25^+^foxp3^−^ cells, which presented higher frequencies of BV6^+^ cells compared to other specificities in F1 mice (fig. 4D), or to BV6^+^ cells of B6 mice (fig S3C). Likewise, these increased frequencies translated into high numbers of these cells, equal to the numbers of cells carrying other specificities (fig. 4E), again showing a lack of physical depletion of Sag-specific cells at this stage. Remarkably, up to 25% of CD25^+^foxp3^+^ cells could be Mls-1^a^-specific at day 3 of life of F1 mice. The Treg repertoire normalized thereafter (fig. S4). Thus, our results demonstrate that early expression of the self-antigen Mls-1^a^ resulted in negative selection of BV6^+^CD4^+^ cells whereas nTreg of the same specificity appeared to “resist” this process.

In order to determine whether the strength of the TCR signal would impact on STAT-5 sensitivity, we analyzed whether more BV6^+^ cells would activate STAT-5 in response to the Sag. We did not observe such a correlation: similar frequencies of pSTAT-5^+^ cells were found in all CD4^+^CD8^−^ subsets defined by CD25 and foxp3 expression when BV6^+^ and BV6^−^ cells were compared at day 3 after birth (Table 1). Notably, a similar proportion of CD25^+^foxp3^+^ cells expressed pSTAT-5 in BV6^+^ and BV6^−^ cells although the overall proportion of CD25^+^foxp3^+^ cells was greatly enhanced in BV6^+^ cells (fig. 4B). Thus, the proportion of Sag-specific nTreg cells activating STAT-5 was independent of the selection of a greater number of cells into the regulatory lineage.

**Table 1:** Self-specific nTreg are not enriched in pSTAT-5^+^ cellsa).

	CD25^−^foxp3^−^	CD25^−^foxp3^+^	CD25^+^foxp3^−^	CD25^+^foxp3^+^
BV6^−^	1.4±1.0	10.8±4.25	7.7±5.0	31.9±2.0
BV6^+^	1.5±0.9	11.8±10.5	3.6±2,7	32,7±7,5

a) Results shown are the percentages of pSTAT-5^+^ cells in BV6^+^ and BV6^−^ CD4^+^CD8^−^ cells (rows) within cells of the indicated phenotype (columns) in the thymus of C57BL/6×DBA/2 mice at day 3 after birth. Results are compiled from 2 independent experiments from 6 mice.

## Discussion

Our results demonstrate that pSTAT-5 and foxp3 are closely linked during CD4^+^CD25^+^foxp3^+^ T cell differentiation in un-manipulated mice. We show that pSTAT-5 is associated with foxp3 expression in adult mice and during appearance of nTreg cells in the first week of life. This association is however not as simple as one might have predicted from the recent data showing a role for STAT-5 in inducing foxp3 in the thymus and in the periphery [27], [28], [30]. Notably, we did not find enrichment in the frequency of pSTAT-5^+^ cells in CD25^+^foxp3^−^ precursors of nTreg cells during ontogeny, a result that would have been expected if one postulate that STAT-5 activation precedes foxp3 expression. Rather, we observed that pSTAT-5^+^ cells represented only a minor subset of CD25^+^foxp3^−^ precursors in frequency. Interestingly, we found similar numbers of pSTAT-5^+^CD25^+^foxp3^−^ cells and of more mature pSTAT-5^+^CD25^+^foxp3^+^ cells during early life of mice, though. If one takes into account the positive effect of pSTAT-5 on foxp3 expression [27], [28], thus, this result suggests that pSTAT-5^+^CD25^+^foxp3^−^ cells might represent the precursors of CD25^+^foxp3^+^ cells. Indeed, it has been shown in two independent settings, that only a fraction of CD25^+^foxp3^−^ cells gave rise to CD25^+^foxp3^+^ cells [28], [30] and in proportions that are consistent with the frequency of pSTAT-5^+^ cells that we report here. Equal numbers of both subsets would suggest that transition from one to another might proceed without cell division, at least at this age. A direct demonstration of this hypothesis will await the technical possibility to sort out live pSTAT-5^+^ cells.

The CD25^+^foxp3^+^ cells expressed the highest levels of CD122 and had the highest proportions of pSTAT-5^+^ cells compared to other subsets *ex vivo*. In contrast to CD25^+^foxp3^+^ mature nTreg cells, the CD25^+^foxp3^−^ subset expressed low levels of CD122 and exhibited low levels of STAT-5 activation *ex vivo*. Moreover, IL-2/IL-15 induced pSTAT-5 preferentially in CD122^hi^ foxp3^+/lo^ cells rather than in CD25^hi^ cells *in vitro* and presumably *in vivo*. In the same line, CD25^−^foxp3^lo^ cells activated STAT-5 at greater levels than CD25^+^foxp3^−^ cells upon *in vitro* stimulation with IL-2 and/or IL-15 and had higher CD122 expression levels than CD25^+^foxp3^−^ cells. Thus, STAT-5 activation *ex vivo* and *in vitro* was associated with CD122 and foxp3 rather than with CD25 expression. What could explain this result? Foxp3 itself might be responsible for the enhanced activation of the IL-2/IL-15 signaling pathway in CD122^+^ cells through repression of SOCS-1 and SOCS-3 expression, known inhibitors of STAT-5 activation [31], [32]. Another possibility is that foxp3 might confer higher susceptibility to IL-2 through a direct effect on CD122 expression. However, foxp3 is not reported to regulate expression of CD122, in contrast to CD25 [33], [34]. In any case, our results question the assumption that CD25 expression by itself predicts susceptibility to IL-2 signaling. Our results confirm in normal mice what was observed in genetically deficient mice, i.e. the more important role of CD122 compared to CD25 in regulating foxp3 expression in the thymus [20], [21], [22], [23].

Interestingly, CD25^−^foxp3^lo^ cells exhibited low levels of pSTAT-5 *ex vivo* but that was not due to a lower sensitivity towards IL-2 since most of the cells were able to activate pSTAT-5 in vitro upon IL-2 stimulation due to a high expression level of CD122. Thus, these cells might be in contact with limited amount of IL-2 *in vivo*. The simultaneous appearance of CD25^−^foxp3^lo^ and CD25^+^foxp3^+^ cells during ontogeny, as shown here, and in foxp3-GFP transgenic neonates [2], suggest that these two populations might somehow be inter-related. This hypothesis is further supported by a great deal of homology in their phenotype [2]. Another experimental evidence in favor of an intimate relationship between CD25^−^foxp3^lo^ and CD25^+^foxp3^+^ cells comes from the observation that CD25^+^foxp3^+^ cells may convert into CD25^−^foxp3^lo^ cells upon limiting amount of IL-2 *in vivo* [35], which is known to directly regulate CD25 and foxp3 expression.

The role of endogenous Sags in shaping the T cell repertoire has fueled many reports [36], including the first detailed description of negative selection in the thymus as a tolerogenic process [37]. Likewise, early studies with endogenous [38] or exogenous [39] Sags reported that Sag-specific T cells were enriched in CD25^+^ T cells, without information on foxp3 expression, unknown at that time. More recently, Ribot et al have shown that endogenous Sags expressed by thymic epithelium efficiently select Sag-specific foxp3^+^ T cells in irradiated bone marrow chimeras [40]. From the results described above, one might have predicted that physiological expression of endogenous Sags would have resulted in the selection of a higher frequency of Sag-specific nTreg cells. Nevertheless, we show here for the first time to our knowledge that expression of an endogenous Sag result in enhanced selection of Sag-specific regulatory T cells early in life. Although selection of antigen-specific regulatory T cells in the thymus upon recognition of the cognate antigen in TCR transgenic mice is a well-established result, our data represent one of the few examples showing enhanced selection of foxp3^+^ cells due to expression of an endogenous self-antigen in the polyclonal repertoire of normal mice. This enhanced selection of self-specific foxp3^+^ T cells will certainly help to decipher signaling events affecting this population during their differentiation.

In that regard, among cells that were committed to the regulatory lineage (i.e. CD25^+^foxp3^−^ and CD25^+^foxp3^+^ cells), we observed that expression of pSTAT-5 was found in similar proportions in BV6^+^ cells and in BV6^−^ cells, showing that pSTAT-5 expression was not restricted to Sag-specific cells but rather affected the nTreg population as a whole. Thus, our results confirm that the TCR signal is the prime factor in shaping the T cell repertoire of foxp3^+^ cells. The ensuing cytokine signal, mediated by IL-2/STAT-5 is independent of the TCR specificity and is probably necessary to “secure” foxp3 expression (although numerous foxp3^+^ cells did not expressed detectable levels of pSTAT-5 by flow cytometry). Because STAT-5 functions as a inducer of Bcl-2 and Bcl-xL [41], which are crucial anti-apoptotic molecules, STAT-5 may also be involved in the survival of some thymocytes recognizing self-antigens. This signal may allow for a sufficient amount of time for the cells to up-regulate foxp3 under the influences of additional signals, such as TCR-induced CARMA-1 leading to c-rel activation [42]. In that respect, it has been suggested that a Bcl-2 transgene can rescue foxp3 expression in STAT-5-KO mice (unpublished observations cited in [9]) but not in CARMA-1 [10], [11] or c-rel-KO mice [42], demonstrating that STAT-5 and foxp3 expression are not absolutely intertwined. It can then be deduced that STAT-5 activation might be more important for promoting survival of autoreactive nTreg cells than to directly induce foxp3 expression in the murine thymus.

## Supporting Information

Figure S1 **STAT5 and pSTAT-5 stainings in CD4^+^CD8^−^ murine thymocytes.** Representative flow cytometry analysis of phosphoryled STAT-5 (pSTAT-5) vs. foxp3 expression in (**A**) freshly isolated C57BL/6 thymocytes *ex vivo*, or (**B**) in splenocytes stimulated 30 min. with IL-2 *in vitro* with or without pre-incubation with a pSTAT-5 blocking peptide spanning the Y694 of STAT-5. Similar results were obtained in an additional independent experiment. (**C**) Shown is the Median Fluorescence Intensity (MFI) of pSTAT5 in pSTAT5+ cells within the indicated CD4+ subsets in the thymus of adult C57BL/6 mice. Each dot corresponds to a single mouse. (**D**) Shown is the Median Fluorescence Intensity (MFI) of STAT5 within the indicated CD4+ subsets in the thymus of a single C57BL/6 mouse determined in triplicates.
(TIFF)

Figure S2 **Evolution of the T cell repertoire during ontogeny of C57BL/6**×**DBA/2 and C57BL/6 mice.** (**A**) *Left panel*: gate defining CD4^+^CD8^−^ cells is shown in the figure. *Right panel*: BV6 expression in CD4^+^CD8^−^ cells of the thymus of a 3-day old B6D2 neonate. Gate used to determine frequencies of BV6^+^ cells is shown in the figure. (**B**) Frequencies of BV6^+^, BV11^+^, and BV8.1/8.2^+^ in CD4^+^CD8^−^ thymocytes determined by flow cytometry are shown at the indicated days after birth in the thymus of C57BL/6×DBA/2 (B6D2) and C57BL/6 (B6) mice. Results shown are compiled from 17 independent experiments.
(TIFF)

Figure S3 **TCR repertoire of thymic CD4^+^CD8^−^ cells in subsets defined by CD25 and foxp3 expression in C57BL/6 neonates.** (**A–D**) Frequencies and absolute numbers of CD25^+^foxp3^+^ cells (**A, B**) and of CD25^+^foxp3^−^ cells (**C, D**) expressing the indicated TCR in C57BL/6 (B6) mice at the indicated ages after birth. Results shown are compiled from 17 independent experiments.
(TIFF)

Figure S4 **T cell repertoire analysis in CD4^+^CD8^−^ cells defined by CD25 and foxp3 expression in C57BL/6**×**DBA/2 and C57BL/6 neonates.** (**A–D**) Frequencies of (**A,C**) CD25^+^foxp3^−^ or (**B, D**) CD25^+^foxp3^+^ cells expressing the indicated TCR in C57BL/6×DBA/2 (B6D2) or C57BL/6 (B6) mice at the indicated ages after birth. Results shown are compiled from 17 independent experiments.
(TIFF)
