# Supplementary figures and images for: Continuous Activation of the CD122/STAT-5 Signaling Pathway during Selection of Antigen-Specific Regulatory T Cells in the Murine Thymus
Source: PLoS One. 2011 Apr 26;6(4):e19038. doi: 10.1371/journal.pone.0019038 (PMC3082544; doi:10.1371/journal.pone.0019038)

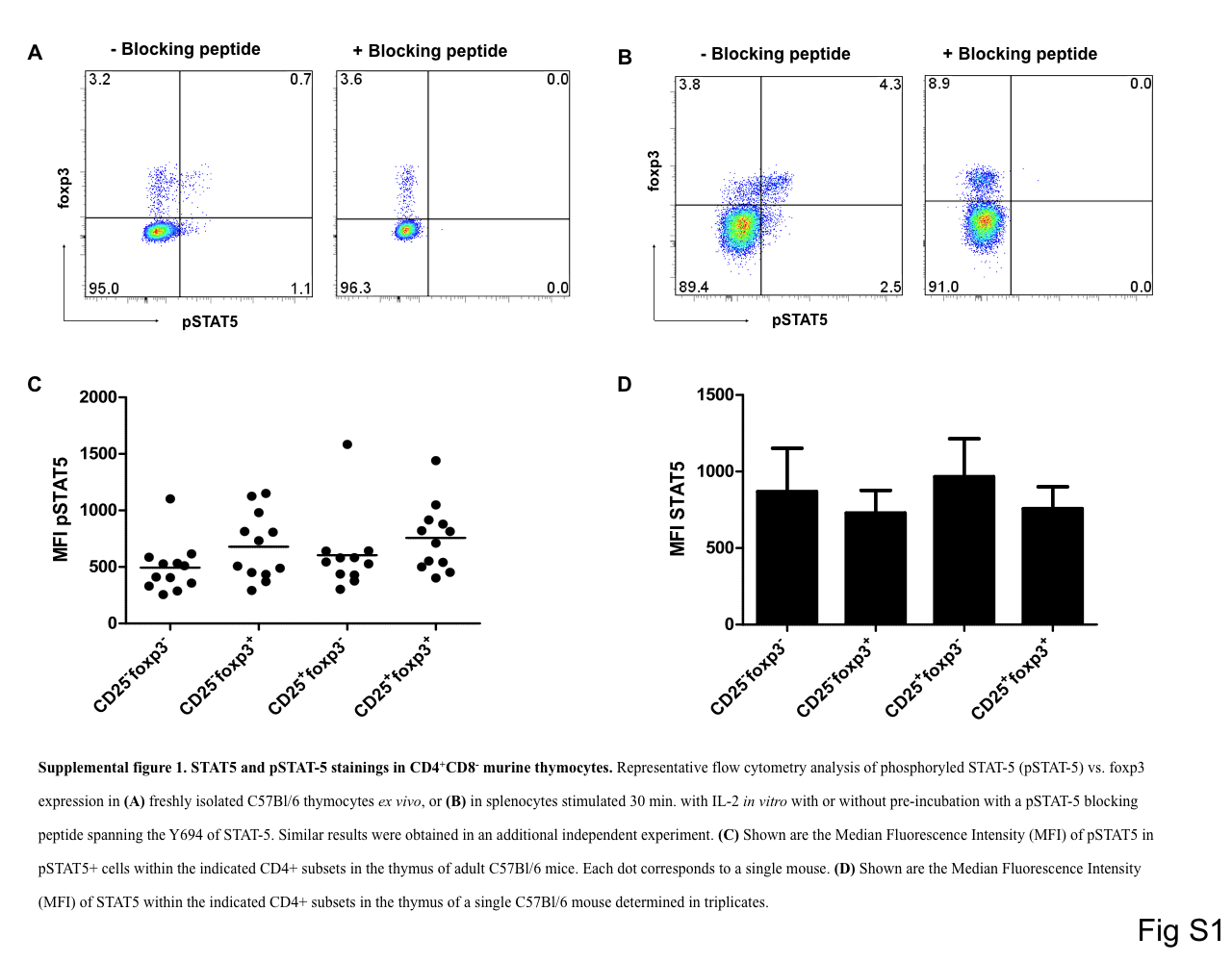

Supplement: Figure S1 — STAT5 and pSTAT-5 stainings in CD4+CD8− murine thymocytes. Representative flow cytometry analysis of phosphoryled STAT-5 (pSTAT-5) vs. foxp3 expression in (A) freshly isolated C57BL/6 thymocytes ex vivo, or (B) in splenocytes stimulated 30 min. with IL-2 in vitro with or without pre-incubation with a pSTAT-5 blocking peptide spanning the Y694 of STAT-5. Similar results were obtained in an additional independent experiment. (C) Shown is the Median Fluorescence Intensity (MFI) of pSTAT5 in pSTAT5+ cells within the indicated CD4+ subsets in the thymus of adult C57BL/6 mice. Each dot corresponds to a single mouse. (D) Shown is the Median Fluorescence Intensity (MFI) of STAT5 within the indicated CD4+ subsets in the thymus of a single C57BL/6 mouse determined in triplicates. (TIFF) [file pone.0019038.s001.tiff]

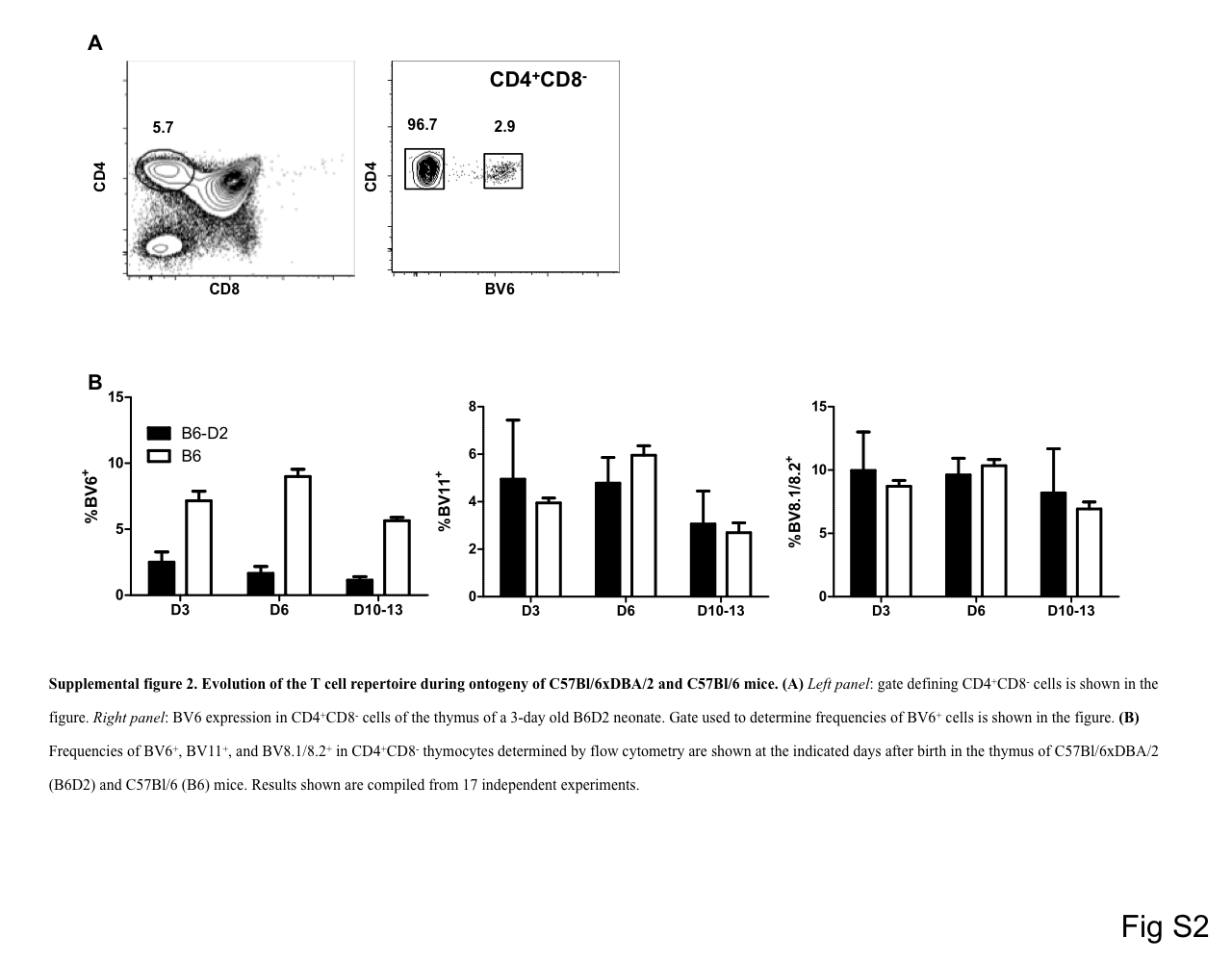

Supplement: Figure S2 — Evolution of the T cell repertoire during ontogeny of C57BL/6×DBA/2 and C57BL/6 mice. (A) Left panel: gate defining CD4+CD8− cells is shown in the figure. Right panel: BV6 expression in CD4+CD8− cells of the thymus of a 3-day old B6D2 neonate. Gate used to determine frequencies of BV6+ cells is shown in the figure. (B) Frequencies of BV6+, BV11+, and BV8.1/8.2+ in CD4+CD8− thymocytes determined by flow cytometry are shown at the indicated days after birth in the thymus of C57BL/6×DBA/2 (B6D2) and C57BL/6 (B6) mice. Results shown are compiled from 17 independent experiments. (TIFF) [file pone.0019038.s002.tiff]

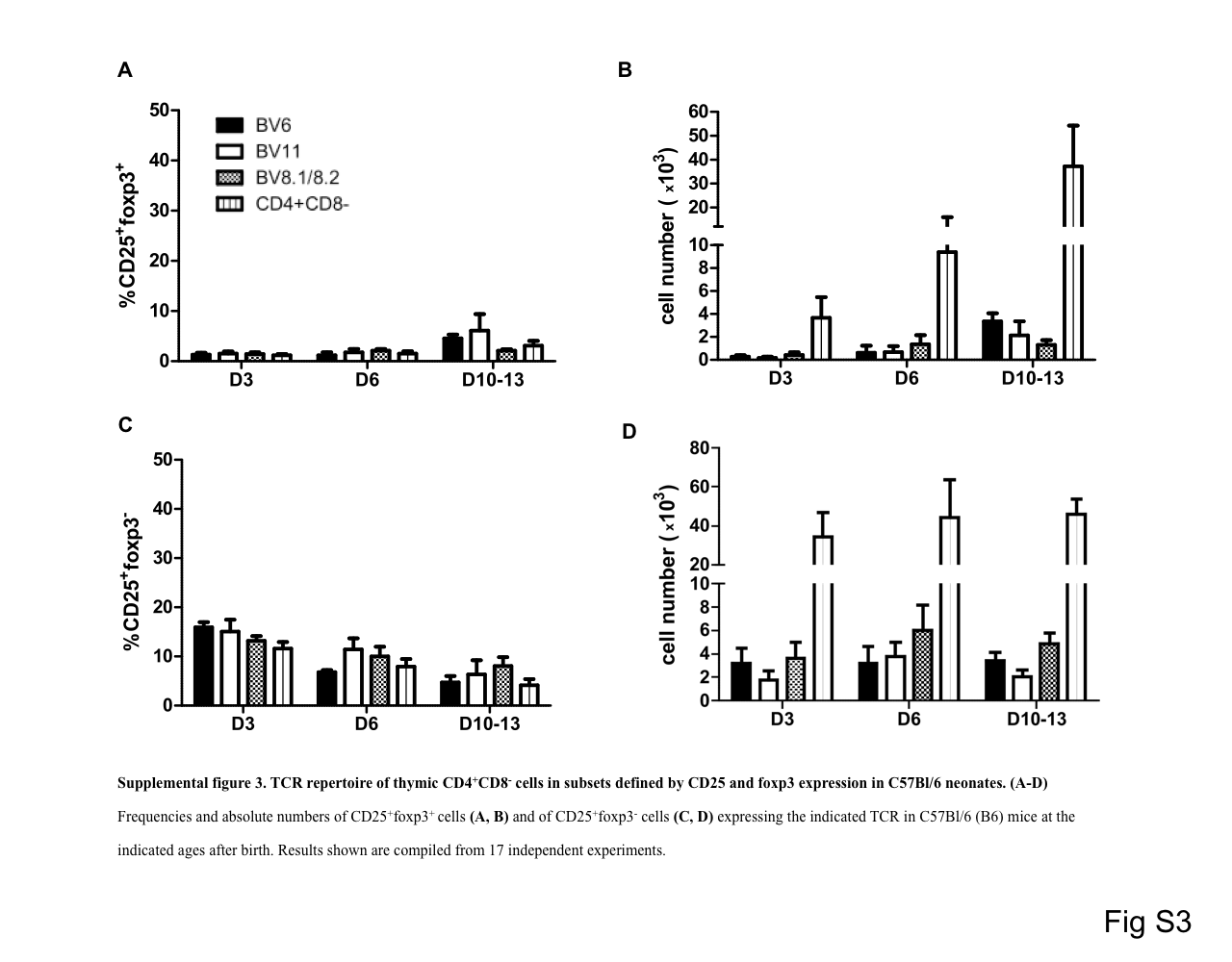

Supplement: Figure S3 — TCR repertoire of thymic CD4+CD8− cells in subsets defined by CD25 and foxp3 expression in C57BL/6 neonates. (A–D) Frequencies and absolute numbers of CD25+foxp3+ cells (A, B) and of CD25+foxp3− cells (C, D) expressing the indicated TCR in C57BL/6 (B6) mice at the indicated ages after birth. Results shown are compiled from 17 independent experiments. (TIFF) [file pone.0019038.s003.tiff]

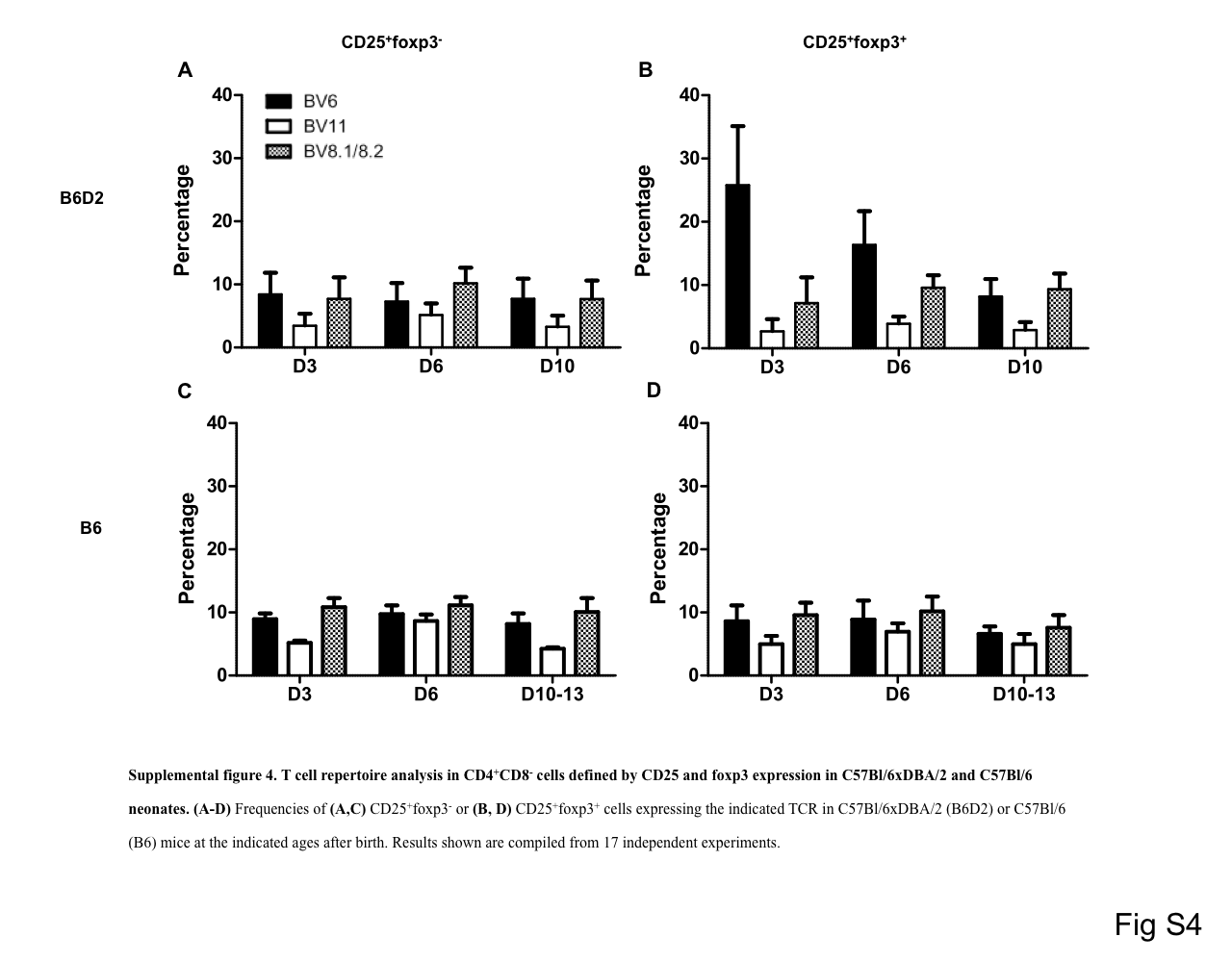

Supplement: Figure S4 — T cell repertoire analysis in CD4+CD8− cells defined by CD25 and foxp3 expression in C57BL/6×DBA/2 and C57BL/6 neonates. (A–D) Frequencies of (A,C) CD25+foxp3− or (B, D) CD25+foxp3+ cells expressing the indicated TCR in C57BL/6×DBA/2 (B6D2) or C57BL/6 (B6) mice at the indicated ages after birth. Results shown are compiled from 17 independent experiments. (TIFF) [file pone.0019038.s004.tiff]
